# Prevalence and predictors of intra-abdominal hypertension and compartment syndrome in surgical patients in critical care units at Kenyatta National Hospital

**DOI:** 10.1186/s12873-017-0120-y

**Published:** 2017-03-23

**Authors:** A. Muturi, P. Ndaguatha, Daniel Ojuka, A. Kibet

**Affiliations:** 1 University of Nairobi, P. O Box 14523–00800, Nairobi, Kenya; 2 Department of Surgery, University of Nairobi, P. O Box 30197, Nairobi, 00100 Kenya; 3 Department of Anaesthesia and Critical Care, Kenyatta National Hospital, P. O. Box 20723-00202 Nairobi, Kenya

**Keywords:** Intraabdominal pressure, Intraabdominal hypertension, Abdominal compartment syndrome

## Abstract

**Background:**

Intra-abdominal hypertension (IAH) affects almost every organ sytem.If it is not detected early and corrected, mortality would be high. The prevalence of IAH and abdominal compartment syndrome (ACS) at Kenyatta National Hospital (KNH) critical care units is not known. The aim of this sudy was to determine the prevalence and factors associated with development of IAH/ACS among critically ill surgical patients.

**Methods:**

This was a cross sectional descriptive study involving surgical patients in critical care units at KNH, carried out from March 2015 to October 2015.

One hundred and thirteen critically ill and ventilated patients 13 years or older were recruited into the study.

Krohn’s intravesical method was used to measure intra- abdominal pressure (IAP). Measurements were done at first contact, then at 12 and 24 h. Additional parameters recorded included: laboratory tests such as serum bilirubin and total blood count as well as clinical parameters such as urine output, vital signs and peak airway pressure, among others.

Frequency, means and standard deviation were used to describe the data. Categorical variables e.g. age, were analysed using Chi square test and continous variables using student ‘t’ test and Mann Whitney test as appropriate

**Result:**

A total of 113 consecutive surgical patients admitted to the critical care units were recruited. Of our study population, 71.7% (by IAP max) and 67.3% (by IAP mean) had IAH. Abdominal compartment syndrome (ACS) developed in 4.4% of the population. The following factors were significant determinants of risk of IAH : amount of IV fluids over 24 h (3949.6 vs 2931.1, *p* = 0.003, adjusted OR 1.0 [1.0-1.002]), haemoglobin values at admission (9.9 vs 12.0, *p* = <0.012, adjusted OR 0.6 [0.4-0.9]), peak airway pressure (28.4 vs 17.3; *p* = 0.018, adjusted OR 1.6 [1.1-2.4]) and synchronised intermittent mandatory ventilation (SIMV) (60 vs 32; *p* = 0.041, adjusted OR 1.4 [0.78-2.04]).

Of those who had IAH; age, amount of iv fluids over 24 h, fluid balance and ventilator mode were significant determinants of risk of progression to ACS .

**Conclusion:**

The prevalence of intraabdominal hypertension and abdominal compartment syndrome at KNH is high. Clinical parameters pertaining to fluids administration and ventilator mode are siginificant determinants.

**Electronic supplementary material:**

The online version of this article (doi:10.1186/s12873-017-0120-y) contains supplementary material, which is available to authorized users.

## Background

Intraabdomninal hypertension (IAH) refers to elevated intraabdominal pressure (IAP) >12 mmHg, while abdominal compartment syndrome(ACS) is defined as IAP >20 mmHg with atleast one new organ dysfunction [1]. The prevalence of IAH among critically ill patients is reported to be as high as 50% [2]. It is an independent predictor of organ dysfunction, multiple organ failure and morbidity with a high mortality rate in the absence of prompt and adequate treatment [2, 3]. The predisposing factors include conditions that results in reduced abdominal wall compliance, increased abdominal contents, and increased capillary leakage and fluid resuscitation [4]. These causes reduced cardiac output, restricted chest wall compliance, reduced visceral perfusion and lead to elevated intracranial pressure [4]. Intraabdominal hypertension and ACS can be prevented by regular measurement of IAP, and optimising physiological parameters such as fluid balance, acid–base status, haemodynamic status, respiratory parameters among other factors [4–6]

Krohn’s method of measuring IAP is the most widely used and it utilises indwelling urethral catheter connected to either a transducer or a saline manometer [7]. It is preferred because it is relatively non-invasive, simple and reproducible [7, 8].

IAH still remains largely under diagnosed and unreported, given that in most critical care units (CCUs) IAP is not routinely measured [9]. Previous studies had both medical and surgical patient population, used different definitions and different IAP measurement techniques [10]. To our knowledge, few studies on prevalence of IAH and ACS among surgical patients have been done in Africa. A prospective cohort study of 38 critically ill postlaparotomy patients in Zimbabwe, found prevalence of IAH of 92% and ACS at 8% [11]. Another prospective study, involving 192 paediatric and adult patients in general surgical wards in Uganda, reported IAH prevalence of 25 and 18.4% for paediatric and adult population, respectively [12].

Understanding the frequency and risk factors of IAH and ACS among surgical patients in critical care may lead to early recognition and timely intervention and thus improved outcomes. Using the established World Society of Abdominal Compartment Syndrome (WSACS) 2013 consensus statement on definitions and Krohns measurement technique, we conducted an observational study to determine the prevalence and possible predictors for IAH and ACS among surgical patients in critical care units, across surgical specialities at Kenyatta National Hospital.

## Methods

### Study design

This was a prospective cross sectional study that was conducted in 7 months from march 2015 to October 2015.

### Study site

The study site was KNH, intensive care units (ICUs): Main ICU, cardiac ICU, Neurosurgery ICU, Burns unit and Accident & Emergency department ICU.

### Study population

Patients being cared for by the surgical team admitted in the various critical care units.

The patients were broadly categorised into two: those with abdominal pelvic diagnosis and those whose pathology or disease entity affected other areas of the body, that is, non-abdominal pelvic diagnosis. Those with abdominopelvic conditions would generate data on primary IAH and consequently primary ACS while those with non-abdominopelvic diagnosis expected to have secondary IAH and ACS.

### Inclusion criteria

The following cases were considered eligible for inclusion in the study:

1. ≥13 years and older
2. Surgical patients admitted in the critical care units, intubated and on mechanical ventilation.
3. Patients whose next kin or guardian consented for them to participate in the study

For the purpose of this study, a surgical patient was defined as one who based on the diagnosis, would have been admitted to the general surgical, orthopaedic or any of the speciality surgical units i.e. neurosurgery, cardiothoracic and plastic surgery units, were it not for the critical nature of their illness. This excluded gynaecological and obstetric patients

### Exclusion criteria

The following were excluded from the study:

1. Patients with suprapubic catheter.
2. Patients already known to have bladder outlet obstruction e.g. from benign prostatic enlargement.
3. Patients with burst abdomen or those who have already undergone damage control laparotomy and temporary abdominal closure(TAC) before admission to KNH CCU

### Sampling method

One hundred and thirteen patients who met the inclusion criteria and their kin consented for them to take part in the study were included. Consecutive sampling was used.

### Data collection

The study commenced once approved by the department of surgery and Ethical Research Committee (ERC) - KNH/UON. I, the principal investigator was assisted by two research assistants who were at the level of general surgical resident in clinical rotations. Those who agreed for their kin to participate in the study, informed written consent was obtained and subsequently enrolled in the study. Information obtained included bio data, diagnosis, clinical parameters such amount of fluids administered, pints of blood given, fluid balance and vital signs (blood pressure,temperature, pulse and respiratory rate) laboratory tests (white cell count, haemoglobin, bilirubin, urea and creatinine) IAP at first contact, IAP at 12 h and IAP at 24 h. Only data from patients who had all the clinical, laboratory parameters and all three IAP measurements completed was included in the final analysis.

### Measurement of intra-abdominal pressure

The abdominal pressure was determined using indirect method whereby urinary bladder pressure is measured with a Foley’s catheter. Patients were catheterized with a 16-gauge two- way Foley’s catheter, bladder drained and then filled with 25 cc of sterile saline through the Foley’s catheter. The tubing of the collecting bag were clamped and catheter connected to a saline manometer using two three-way B-BRAUN™ stopcocks connected in series . With the patient in a supine position with abdominal muscles relaxed, the point along the midaxillary line at the level of anterior superior iliac spine was used as the zero reference point. IAP was then measured in centimetres of water at end-expiration 30–60 s after instillation of the priming 25 cc of saline into the bladder. A conversion factor of 1.36 was used to convert the pressure into millimetres of Hg.

Based on IAP, intraabdominal hypertension was graded as follows:

- Grade 0 < 12mmhg
- Grade 1 12-15mmhg
- Grade 2 16-20mmhg
- Grade 3 21-25mmhg
- Grade 4 > 25mmhg

Abdominal compartment syndrome was defined as a sustained IAP >20 mm Hg (with or without APP < 60 mmHg) that was associated with new organ dysfunction/failure.

### Patient care

Those with grade 2–4 IAH were recommended for non surgical interventions to reduce IAP and those with ACS decompressive laparotomy.

#### Data analysis

Intra-abdominal pressure, number of patients with IAH and number of patients with ACS were taken as the independent variables while the clinical and laboratory parameters listed above were the dependant variables.

The data was analyzed using Statistical Package for Social Sciences (SPSS) for Windows Version 21 (Chicago 3).

Measures such as frequency, mean and standard deviation were used to describe the data . Correlates of elevated IAP were determined using Chi square test for categorical variables and Student ‘t’ test and Mann Whitney for continuous variables as appropriate. Univariate and multivariate analysis and logistical regression were then used to correlate IAP to the statistically significant factors with *p* value set at <0.05.

### Ethical considerations

The study commenced upon KNH/UoN ERC approval. At completion of the study, raw data on hard copy was destroyed.

#### Feedback of information

All participants next of kin were informed of the IAP measurements and further care needed depending on the severity.

## Results

### Characteristics of the study population

**Fig. 1:**
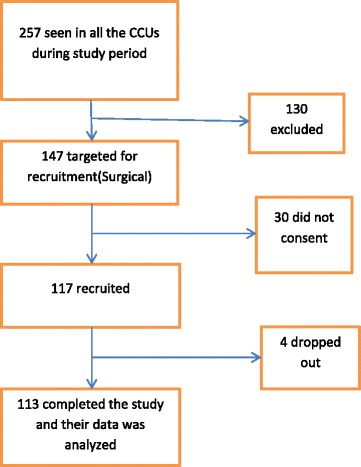
Recruitment scheme. During the study period a total of 257 patients(across all specialities) were seen at the CCUs. Those who met inclusion criteria were 147,out of which 117 had consent given to take part in the study. Four withdrew consent halfway through the study. A total of 113 patients who had the three IAP measurements taken and had the laboratory results were analysed

Of the 113 patients analysed, 70. 8% were male, ranging in age from 15 to 90 years with a mean of 37.2 years (Table 1), (Fig. 1) and (Additional file 1).

**Table 1:** Sociodemographic,clinical and laboratory data

Variable	Frequency (%)	
Gender		
^a^Male	80 (70.8)	
Female	33 (29.2)	
Age in years		
^b^Mean (SD)	37.2 (12.8)	
Min-Max	15–90	
Clinical and laboratory parameters		
Variable	Mean (SD)	Min-Max
^c^Amount of IV fluids over 24 h in ML	3616.1 (1416.8)	1800–6900
Urine output in 24 h	1949.9 (598.3)	800–3800
^d^Fluid balance over 24 h	1698.8 (1368.1)	100–8500
Number of pints of blood transfused over 24 h	1.4 (1.6)	0–6
Pulse rate	101.9 (31.4)	55.3
Systolic Blood Pressure	105.7 (20.4)	56.0
Respiratory rate	22.8 (10.3)	11.0
^e^PAP	18.4 (4.1)	6.0
Temperature	36.1 (5.3)	3.0
^f^WBC	11.8 (9.9)	3.4
^g^Ventilation mode, n (%)		
bipap	5 (4.4)	
cpap	16 (14.2)	
simv	92 (81.4)	
Amount of positive end expiration pressure (PEEP) administered	4.5 (0.7)	4–8
Haemoglobin	10.6 (3.0)	4.1–21.9
Platelet count	321.7 (117.1)	4.1–791.0
Serum creatinine	118.6 (70.8)	4.0–723.0
Serum urea	10.6 (11.0)	2.3–87.0
Serum bilirubin	18.0 (12.8)	5.2–36.0
Base excess	−2.5 (6.2)	−26.5–9.4
^h^Prevalence and grade of IAH and ACS		
Variable	Mean IAP in 24 h Frequency (%)	Hours frequency (%)
Grade 0	37 (32.7)	32 (28.3)
Grade 1	28 (24.8)	22 (19.5)
Grade 2	29 (25.7)	32 (28.3)
Grade 3	13 (11.5)	19 (16.8)
Grade 4	6 (5.3)	8 (7.1)
IAH	76 (67.3)	81 (71.7)
ACS	5 (4.4)	
Primary and secondary IAH based on diagnosis at admission		
^i^pathology	*N* = 76 using IAP mean(%) Frequency (%)	*N* = 81using IAP max(%)
Abdominal pelvic(primary)	30 (39.5%)	33(40.7%)
Non abdominal pelvic(secondary)	46 (60.5%)	48(59.3%)

Presented as frequencies, mean and standard deviations

^a^Majority of the patients were male 70.8%

^b^The mean age of the study population was 37.2 years

^c^ The amount of intravenous fluids administered over 24 h as recorded in input–output chart

^d^ The fluid balance was derived from the difference of the total amount of fluids administered(sum of IV fluids and enteral and parenteral feeds) and the output(urine output plus 700 ml of estimated insensible fluid losses)

^e^ Peak airway pressure (PAP) in cm of H20 as displayed on the ventilator

^f^ White cell count (WBC) one of the parameters from total blood count profile others considered being haemoglobin and platelets count

^g^Ventilation mode as set by the intensive care team. Biphasic positive airway pressure (Bipap), Continuous airway pressure (CPAP), Synchronised intermittent mandatory ventilation(SIMV)

^h^Of the 113 patients analysed,76(67.3%) had intraabdominal pressure (IAH) when the mean intraabdominal pressure in 24 h was considered. This number rose to 81(71.7%) when the maximal (highest reading in 24 h) is considered. The IAH was categorised in severity from most mild (grade 0, no IAH) to most severe level (grade 4) based on the intrabadominal pressure readings in mm Hg after conversion from cm of H20

^i^The patients were categorised based on diagnosis at admission into those whose primary pathology was in the abdominal pelvic region and the others to be able to generate data on primary(of those with adominopelvic conditions) and secondary(those with other [non abdominopelvic] conditions) IAH and ACS.When mean IAP is considered, of those who developed IAH, 60.5% had non abdominopelvic conditions therefore secondary IAH. This number is similar when maximal IAP is considered

To be able to estimate the magnitude of IAH and ACS, three IAP measurements were done, that is, at admission (baseline), at 12 h and at 24 h. From these measurements, maximal (highest in 24 h) and mean IAP were recorded. Using mean IAP, the number of patients considered to have IAH were 76 (67.3%).The prevalence rose to 81 (71.7%), when maximal IAP was considered. Of the 113, five were deemed to have ACS-based on presence of severe IAH and documented organ failure. This gives ACS prevalence of this group as 4.4%. Of those who had IAH, 39.5% (using IAP mean) and 40.7% (using IAP max) had primary IAH. Considering IAP mean, 60.5% had secondary IAH and the figure is similar at 59.3% when IAP max is considered (Table 1).

Of the five patients who met the criteria for ACS, 4 (80%) had primary ACS and 1 (20%) had secondary ACS.

**Fig. 2:**
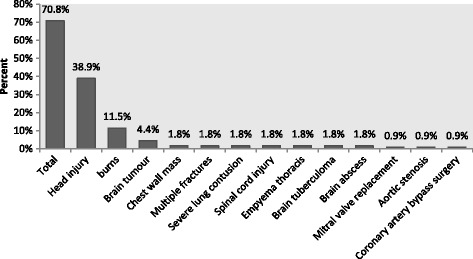
Patients with non- abdominopelvic (secondary) causes of IAH/ACS. On the x axis the bars represents the diagnosis categories as recorded at admission to critical care unit. Of the 113 patients analysed 70.8% had non- abdominopelvic conditions and the specific disease entities are enumerated. The Y axis has the proportion of those with non -abdominopelvic conditions in % out of the total of 113

Of the 113 patients, only 29.2% had abdomino-pelvic conditions. Majority had non-abdominal pelvic conditions (Fig. 2).

When mean IAP is considered,the following parameters were found to be significant determinants of risk of IAH:amount of iv fluids in 24 h, number of pints of blood transfused in 24 h, ventilation mode, peak airway pressure and haemoglobin level. Upon multivariate logistic regression, the following parameters remained significant: Hb, peak airwy pressure, amount of fluids in 24 h and SIMV mode of ventilation (Table 2).

**Table 2:** Factors associated with development of IAH when mean IAP is considered

	IAH		*P* value	Adjusted OR (95% CI)	*P* value
	Yes	No			
Gender					
Male	51 (64.6)	28 (35.4)	0.351	2.2 (0.5–10.3)	0.334
Female	25 (73.5)	9 (26.5)			
Age in years	38.0 (13.3)	35.4 (11.6)	0.316	1.04 (0.98–1.10)	0.249
^a^Amount of IV fluids over 24 h in ML	3949.6 (1431.5)	2931.1 (1121.8)	**0.003**	1.0 (1.0–1.002)	**0.030**
Number of pints of blood transfused over 24 h	1.5 (1.7)	0.5 (1.2)	**0.003**	1.04 (0.59–1.80)	0.904
Fluid balance over 24 h	1992.9 (1454.0)	1094.6 (927.6)	**0.001**	1.0 (1.0–1.002)	0.907
^b^Peak airway pressure 24 h	28.4 (1.7)	17.3 (1.9)	0.008	1.6 (1.1–2.4)	**0.018**
Ventilation mode(%)					
Bipap	1(20.0)	4(80.0)	**0.039**	1.23(0.8–1.69)	0.218
Cpap	15(93.8)	1(6.3)	**0.015**	1.1(1.0–1.22)	0.328
^c^SIMV	60(65.2)	32(34.8)	**0.034**	1.4(0.78–2.04)	**0.041**
Base excess	−3.2 (7.2)	−1.0 (3.1)	0.085	1.10 (0.94–1.28)	0.230
WBC	12.2 (7.8)	11.1 (13.2)	0.606	1.01 (0.97–1.07)	0.505
^d^Hb	9.9 (3.2)	12.0 (1.9)	<0.001	0.60 (0.40–0.90)	**0.012**
Platelets	332.8 (128.0)	294.5 (90.3)	0.109	1.01 (1.00–1.02)	0.661

Categorical data analysed using Chi square and continuous data ‘Mann Whitney U’ test and student ‘t’ test. *P* value <0.05

^a^amount of iv fluids administered over 24 h period

^b^the peak airway pressure incm H20 as displayed on the ventilator

^c^the SIMV ventilation mode

^d^the haemoglobin levels in g/dl

When the maximal IAP is considered, the following parameters were found to be significant determinants of risk of IAH: amount of iv fluids in 24 h, haemoglobin level and fluid balance. Upon multivariate logistic regression, the following parameters remained significant: amount of fluids in 24 h and Hb (Table 3).

**Table 3:** Factors associated with development of IAH when maximal IAP is considered

Variable	IAH		*P* value	Adjusted OR (95% CI)	*P* value
	Yes	No			
Gender					
Male	54 (68.4)	25 (31.6)	0.231	1.0 (0.2–4.5)	0.954
Female	27 (79.4)	7 (20.6)			
Age in years	38.3 (13.3)	34.4 (11.2)	0.150	1.05 (1.00–1.12)	0.076
^a^Amount of IV fluids over 24 h in ML	3861.4 (1435.0)	2995.3 (1176.1)	**0.003**	1.00 (1.00–1.001)	**0.025**
Number of pints of blood transfused over 24 h	1.4 (1.7)	0.5 (1.2)	0.10	0.96 (0.57–1.62)	0.881
Fluid balance over 24 h	1914.3 (1443.0)	1153.1 (979.5)	**0.007**	1.00 (1.00–1.001)	0.797
Peak airway pressure 24 h	27.2 (1.9)	17.5 (1.7)	0.135	1.2 (0.9–1.7)	0.253
Ventilation mode(%)					
Bipap	4(26.0)	1(33.0)	0.139	1.03(0.6–1.59)	0.308
Cpap	22(73.8)	2(6.0)	0.241	1.1(0.97–1.20)	0.151
SIMV	44(55.2)	27(30.7)	0.444	1.0(0.78–2.24)	0.607
Base excess	−2.9 (7.1)	−1.3 (3.1)	0.226	1.1 (1.0–1.3)	0.183
WBC	11.9 (7.6)	11.5 (14.2)	0.843	1.01 (0.95–1.06)	
^b^Hb	10.0 (3.1)	12.1 (2.0)	**0.001**	0.60 (0.41–0.87)	
Platelets	331.6 (125.1)	291.4 (93.3)	0.108	1.01 (1.00–1.02)	

Categorical data analysed using Chi square and continuous data ‘Mann Whitney U’ test and student ‘t’ test. *P* value <0.05

^a^The amount of iv fluids given over 24 h

^b^Haemoglobin level in gram per decilitre

In contrast; gender, age, maximal peak airway pressure, base excess, white cell count and platelets were not significant determinants of IAH.

#### Abdominal compartment syndrome(ACS)

Of the 113 patients sampled, five met the criteria for ACS in that they had severe IAH and atleast one organdysfunction/failure. This represents a prevalence of 4.4%.

This was a small but heterogenous group of patients with the following diagnosis/clinical impression: a middle aged man who was admitted following repair of ruptured slow leaking abdominal aortic aneurysm, a patient with acute pancreatitis with severe sepsis, one with extensive third degree burns, a polytrauma patient with missed blunt abdominal trauma and an elderly lady admitted after colectomy for gangrenous sigmoid volvulus.

All five patients had variable degree of multiple organ dysfunction/failure as evidenced by haematological profile, blood biochemistry and ventilator requirements. The team in the appropriate critical care unit was notified of the high IAP readings and suspicion for ACS. Non surgical interventions including insertion or repositioning of nasogastric tube, insertion of flatus tube, careful titration of IV fluid requirements and appropriate adjustments of ventilator settings. All five showed only modest response to non-surgical interventions. Four had decompressive laparotomy with delayed definitive abdominal wall closure and made full recovery. The burns patient died before the decompressive laparotomy could be performed.

When mean IAP is considered, of those with IAH; age, ventilator mode, amount of IV fluids in 24 h and fluid balance determined risk of progression to ACS (Table 4).

**Table 4:** Factors associated with risk of progression of IAH to ACS

Variable	ACS	IAH	*P* value
Gender			
Male	4 (80.0%)	51 (66.2%)	1.000
Female	1 (20.0%)	26 (33.8%)	
Ventilation mode			
bipap	2 (40.0%)	1 (1.3%)	0.149
cpap	3 (60.0%)	12 (15.6%)	*0.441*
^a^simv	0 (0.0%)	64 (83.1%)	**0.041**
^b^Age in years	53.2 (7.6)	38.5 (13.4)	***0.018***
^c^Amount of IV fluids over 24 h in ML	5800 (5700–6200)	3500 (2700–4900)	***0.005***
Fluid balance over 24 h	2100 (1900–3800)	1300 (900–2700)	*0.051*
Number of pints of blood transfused over 24 h	2 (2–2)	0 (0–2)	0.324
Peak airway pressure 24 h	18 (16.5–20.5)	19 (17–19)	0.942
White blood cell count	12.1 (10.7–13.4)	10.4 (8.0–13.8)	0.783
Haemoglobin	9.3 (8.4–9.7)	10.4 (7.4–11.9)	0.651
Platelet count	112 (94–163)	313 (287–401)	0.191
Base excess	2.0 (−8.6–2.1)	−2.4 (−7.4–2.3)	0.807

Categorical data analyzed using Chi square and continuous data ‘Mann Whitney U’ test and student ‘t’ test. *P* value <0.05

^a^Of the ventilation modes, synchronised intermittent mandatory ventilation(SIMV) was significantly associated with progression of IAH to ACS

^b^Of those with IAH, the ones who progressed to ACS were significantly older than the rest

^c^similar to IAH, amount of IV fluids in 24 h was a significant determinant IAH progressing to ACS

## Discussion

The main aims of this study were to document the prevalence of intraabdominal hypertension and abdominal compartment syndrome and factors significantly associated with development of the same.

The prevalence of IAH and ACS differed depending on whether mean or maximal IAP was used. Mean IAP showed an IAH prevalence of 67.3% and when maximal IAP was considered, 71.7%. Malbrain et al., noted that mean IAP tend to down grade intra-abdominal pressure values and may lead to some cases of IAH and ACS being missed [2] . To improve on accuracy and reliability of mean IAP would require frequent IAP measurements(at least every four hours and more frequent if IAP >12 mmHg) or continous measurement [11, 13]. In the absence of automated IAP measurement devices and in a resource constrained set up, like KNH, that would strain the critical care unit workforce. While maximal IAP may be seen as overdiagnosing IAH and ACS,the overall result is positive in terms of diagnosing and prognosticating these patients [2, 11].

This study revealed an IAH prevalence that is remarkably higher than that quoted in other studies [2, 11]. The prevalence of ACS is however comparable with what is published in literature. Taurai studied a small population of post laparotomy surgical patients in critical care, where among the 38 patients studied,the prevalence of IAH was 57% when considering mean IAP and 60% when maximal IAP was considered, with ACS prevalence stated as 8% [11]. Malbrain et al. carried out the largest multicenter prevalence prospective study of IAH in 13 intensive care units (ICUs) using maximal IAP and found the prevalence of IAH to be 65% and the prevalence of ACS to be 5% among surgical [2]^.^ A possible explation of such a high occurrence of IAH in our study is the fact that due to pressure for bed space in our critical care units, at any given time,the patients in these units are more sick and therefore at a higher risk for IAH than centres with more and bigger CCUs. The prevalence of ACS in our study population may have been higher given that the length of follow up of patients with IAH but not deemed to have ACS was restricted to the 24 h period of monitoring IAP.

Large amount of IV fluids administered over 24 h and the attendant positive fluid balance were significantly associated with development of IAH and ACS. This is in keeping with findings by other investigators [14, 15]^.^ This results from excessive extracellular fluid accumulation within the intestine and the contents there in [16]. This is best avoided by calculating and adhering to individual patient fluid needs and response.

Low haemoglobin and the number of pints of blood transfused to correct the same were positively associated with risk of development of IAH and ACS. A preresuscitation Hb value that is 8 g/dl or less has been reported to be associated with high risk of developing IAH and ACS in acutely ill patients both in the emergency department and in the first 24 h of their care in critical care units [17, 18]. In addition, severely anaemic patients requiring transfusion of at least three pints of packed red cell have high risk of developing IAH and ACS [18]. Although aggressive use of blood and blood products can contribute to fluid overload and cause metabolic acidosis hence worsening the capillary leakage, low crystalloids to blood products ratio help to minimise the total volume of fluid required to restore effective circulating volume [19].

When mean IAP is considered, the subset of patients who had IAH had significantly higher peak airway pressure(PAP) readings compared to those without. This is in keeping with other published work that showed that when considering mean IAP, both peak inspiratory and mean airway pressures are significantly increased in patients with IAH and ACS [20]. Though a positive finding in IAH and ACS, airway pressures do not accurately reflect IAP and cannot be substituted for IAP measurements in patients at risk for IAH/ACS. This is because lung and airway diseases affect peak inspiratory and mean airway pressure [21].

Ventilatory mode had an influence on risk of developing IAH and ACS. SIMV mode was associated with higher odds of developing IAH when mean IAP was considered. Mehrdad et al. reported a significant relationship between ventilation mode and IAP, demonstrating that IAP is mostly affected by SIMV, followed by BIPAP and CPAP in that order [22]. This is explained partly by the finding that pressure support ventilation(PSV) is associated with less IAP elevation and CPAP, BIPAP, SIMV have highest PSV in that decreasing order [23, 24].

In this group, there was no statistically significant difference in base excess between patients with IAH and those without. Base excess and lactate are useful markers for assessing resuscitation adequacy and response among critically ill patients. G. Arabadzhiev et al. evaluated a cohort of 43 surgical patients at risk of IAH and ACS and demonstrated that patients with grade two and grade three IAH had high base excess [25]. Base excess and lactate, as resuscitation end points and biochemical markers of cellular metabolic derangements, have been shown to be useful prognostic indicators in critically ill patients [26].

Abdominal wall is also affected by elevated IAP, in that visceral edema,free intraperitoneal fluid and abdominal packs distend the abdomen leading to decreased abdominal wall compliance [4]. Abdominal wall edema in the setting of shock with attendant aggressive fluid resuscitation also contribute to impaired flexibility [4]. It has been suggested that conditions such as cirrhosis, previous pregnancy and morbid obesity are protective of IAH/ACS since they are associated with increased abdominal wall compliance [4, 27]

The incidence of IAH in patients with severe acute pancreatitis (SAP) is high (60–80% depending on the population considered), with one in three of those with IAH developing full blown ACS,with mortality rate nearly 70% [28]. Factors responsible include: pancreatic and peripancreatic edema (aggravated by excessive IV fluids), ascites, ileus, abdominal wall edema and abdominal pain [29].

Surgery is reserved for those who fail to respond to non surgical therapeutic interventions. It requires prompt recognition of failed medical management which should lead to timely surgical decompression to ensure favourable outcome [30].

Tensely distended abdomen may not be seen in patients with major torso burns with eschar formation. The risk factors for IAH and ACS in major burns patients are:inhalational burns,burns surface 70% or greater, massive fluid resuscitation and deep circumferential torso burns [31]. In these situations, a combination of SIRS, capillary leak and third spacing and extrinsic compression of chest and abdomen by the eschars contribute to development of IAH and ACS [32].

Diuresis, sedation, adequate analgesia, escharotomy and use of colloids may help in mild and moderate cases of IAH, but in ACS decompressive laparotomy is the only treatment option that works [31, 32]. In major burns, routine IAP monitoring is key in preventing IAH and ACS [32, 33].

Polytrauma patients are at risk of ACS from SIRS causing massive capillary leak and third spacing. Another contributing factor is massive blood loss necessitating aggressive resuscitation with Iv fluids and blood transfusion, and intraperitoneal and retroperitoneal bleeding [34]. Even in these critically ill patients, decompressive laparotomy reduces IAP and may also discover major bleeding which can be treated surgically [35]^.^

ACS incidence following open repair of a ruptured AAA is reported to be as high as 30% with a mortality approaching 70% [36]. Massive fluid transfusion,shock at admission and prolonged cross clamp time are recognised risk factors for ACS development. Abdominal decompression is vital to achieve favourable outcome [37]. Routine measurement and early recognition of rising IAP and expedited decompression of the tense abdomen,can lead to mortality reduction after aneurysm repair [38].

Abdominal compartment syndrome following sigmoid colectomy and Hartmann’s colostomy for gangrenous sigmoid volvulus is rare [39].

Though the number of patients who had ACS is small (five), on subgroup analysis, there were significant differences in age, ventilatory mode, amount of iv fluids in 24 h and fluid balance between IAH and ACS groups. Those with IAH who went on to develop ACS, were older, had higher fluid balance, received more iv fluids in 24 h and more were on SIMV ventilatory mode. In a cohort of mechanically ventilated surgical patients, Chok Aik-Yonget showed that advanced age is associated with higher risk of IAH and progression to ACS and poor outcomes [40].

The ventilation mode had an effect on risk of developing both IAH and ACS, with SIMV showing positive correlation. It has been shown that IAP is mostly affected by SIMV, followed by BIPAP and CPAP [22]. Pressure support ventilation (PSV) is associated with less IAP elevation and CPAP, BIPAP, SIMV have highest PSV in that decreasing order [23, 24].

### Study limitation

We studied a fairly heterogenous patient population and because we did not use a scoring/grading system such as APACHE 2 to compare the patients, it is difficult to generalize and make robust conclusions.

I used saline manometer because of lack of transducers. Though this could affect accuracy of measurements, every attempt was made to zero the manometer properly before each IAP measurement.

The study required multiple calibrations and measurements of the IAP. This was mitigated by having the research assistants applying the same technique of zeroing the manometer and measurement for each of the three readings.

Diagnosis of ACS required presence of IAH with at least one organ dysfunction/failure. It was not possible to attribute the organs dysfunction/failure to development of ACS.

We did not perform the full coagulation assessment, only relied on platelet count which is not a full representation of the coagulation status. In addition, we did not relate presence of IAH and ACS with patient outcomes. Something we intend to do in a follow up study.

## Conclusions

In this mixed population of surgical patients, the prevalence of intraabdominal hypertension and abdominal compartment syndrome is high and could be a significant cause of morbidity and mortality. This is due to the deleterious effects of IAH and ACS in virtually all organ systems causing altered organ perfusion and end organ function.

Amount of IV fluids administered over 24 h, fluid balance, haemoglobin levels, high transfusion requirements and SIMV ventilation mode are important determinants of IAH. Of those with IAH, age, amount of IV fluids, fluid balance and ventilation mode seem to predict the risk of progression to ACS.

## Additional file

Additional file 1: Raw data set. Description: Demographic details, laboratory tests, clinical information and IAP measurements. (XLSX 38 kb)

## Abbreviations

ACS: Abdominal compartment syndrome
APP: Abdominal perfusion pressure
BIPAP: Biphasic positive airway pressure
BMI: Body Mass Index
BP: Blood Pressure
BSA: Burnt Surface Area
CCU: Critical care unit
CPAP: Continuous Positive Airway Pressure
CT: Computed tomography
HB: Hemoglobin
IAH: Intra-abdominal hypertension
IAP: Intra-abdominal pressure
IP: Intra-peritoneal
IV: Intravenous
KNH: Kenyatta National Hospital
MAP: Mean arterial pressure
MmHg: Millimeters of mercury
NG: Nasogastric
PSV: Positive pressure ventilation
RAAS: Rennin angiotensin aldosterone system
SD: Standard Deviation
SIMV: Synchronized Intermittent Mandatory Ventilation
SIRS: Systemic Inflammatory Response Syndrome
SPSS: Statistical Package for Social Sciences
UON: University Of Nairobi
WSACS: World society of abdominal compartment syndrome
TAC: Temporary Abdominal Closure
